# Marker-Assisted Introgression of the Salinity Tolerance Locus *Saltol* in Temperate *Japonica* Rice

**DOI:** 10.1186/s12284-023-00619-2

**Published:** 2023-01-12

**Authors:** Caterina Marè, Elisa Zampieri, Viviana Cavallaro, Julien Frouin, Cécile Grenier, Brigitte Courtois, Laurent Brottier, Gianni Tacconi, Franca Finocchiaro, Xavier Serrat, Salvador Nogués, Mireia Bundó, Blanca San Segundo, Noemi Negrini, Michele Pesenti, Gian Attilio Sacchi, Giacomo Gavina, Riccardo Bovina, Stefano Monaco, Alessandro Tondelli, Luigi Cattivelli, Giampiero Valè

**Affiliations:** 1 Council for Agricultural Research and Economics, Research Centre for Genomics and Bioinformatics, Via S. Protaso 302, 29017 Fiorenzuola d’Arda, Piacenza Italy; 2 Council for Agricultural Research and Economics, Research Centre for Cereal and Industrial Crops, s.s. 11 to Torino, km 2.5, 13100 Vercelli, Italy; 3 Institute for Sustainable Plant Protection, National Research Council, Strada Delle Cacce 73, 10135 Turin, Italy; 4 Department of Agricultural and Environmental Sciences - Production, Landscape, Agroenergy - DiSAA, University of Milan, Milan, Italy; 5 CIRAD, UMR AGAP, 34398 Montpellier, France; 6 AGAP, CIRAD, INRAE, Institut Agro, University of Montpellier, Montpellier, France; 7 Departament de Biologia Evolutiva, Ecologia i Ciències Ambientals, Secció de Fisiologia Vegetal, Universitat de Barcelona, Barcelona, Spain; 8 Centre for Research in Agricultural Genomics (CRAG)-CSIC-IRTA-UAB-UB, Bellaterra (Cerdanyola del Vallès), Barcelona, Spain; 9 Consejo Superior de Investigaciones Científicas (CSIC), Barcelona, Spain; 10 SIS Società Italiana Sementi, Via Mirandola, 5, 40068 San Lazzaro di Savena, Bologna Italy; 11 Council for Agricultural Research and Economics, Research Centre for Engineering and Agro-Food Processing, Strada Delle Cacce 73, 10135 Turin, Italy; 12 Dipartimento per lo Sviluppo Sostenibile e la Transizione Ecologica, University of Piemonte Orientale, Piazza S. Eusebio 5, 13100 Vercelli, Italy

**Keywords:** Rice, Breeding, MABC, Salt tolerance, *Saltol*, Background selection, Recovery percentage, KASP markers

## Abstract

**Background:**

Rice is one of the most salt sensitive crops at seedling, early vegetative and reproductive stages. Varieties with salinity tolerance at seedling stage promote an efficient growth at early stages in salt affected soils, leading to healthy vegetative growth that protects crop yield. *Saltol* major QTL confers capacity to young rice plants growing under salt condition by maintaining a low Na^+^/K^+^ molar ratio in the shoots.

**Results:**

Marker-assisted backcross (MABC) procedure was adopted to transfer *Saltol* locus conferring salt tolerance at seedling stage from donor *indica* IR64-*Saltol* to two temperate *japonica* varieties, Vialone Nano and Onice. Forward and background selections were accomplished using polymorphic KASP markers and a final evaluation of genetic background recovery of the selected lines was conducted using 15,580 SNP markers obtained from Genotyping by Sequencing. Three MABC generations followed by two selfing, allowed the identification of introgression lines achieving a recovery of the recurrent parent (RP) genome up to 100% (based on KASP markers) or 98.97% (based on GBS). Lines with highest RP genome recovery (RPGR) were evaluated for agronomical-phenological traits in field under non-salinized conditions. VN1, VN4, O1 lines were selected considering the agronomic evaluations and the RPGR% results as the most interesting for commercial exploitation. A physiological characterization was conducted by evaluating salt tolerance under hydroponic conditions. The selected lines showed lower standard evaluation system (SES) scores: 62% of VN4, and 57% of O1 plants reaching SES 3 or SES 5 respectively, while only 40% of Vialone Nano and 25% of Onice plants recorded scores from 3 to 5, respectively. VN1, VN4 and O1 showed a reduced electrolyte leakage values, and limited negative effects on relative water content and shoot/root fresh weight ratio**.**

**Conclusion:**

The *Saltol* locus was successfully transferred to two elite varieties by MABC in a time frame of three years. The application of background selection until BC_3_F_3_ allowed the selection of lines with a RPGR up to 98.97%. Physiological evaluations for the selected lines indicate an improved salinity tolerance at seedling stage. The results supported the effectiveness of the *Saltol* locus in temperate *japonica* and of the MABC procedure for recovering of the RP favorable traits.

**Supplementary Information:**

The online version contains supplementary material available at 10.1186/s12284-023-00619-2.

## Introduction

Abiotic stresses negatively affect more than 50% of crop yields (Vij and Tyagi 2007) and major losses are caused by salinity (Chinnusamy et al. 2005). At least 33% of arable lands are affected by salinization and more areas are expected to deteriorate in the coming years because of global climate changes (FAO 2016). Indeed, climate change is increasing sea water intrusion in coastal areas and evaporation (www.knowledgebank.irri.org). Moreover, excess of irrigation with incorrect soil drainage, use of poor quality water containing high level of salts and increased capillarity rise of saline groundwater contribute to exacerbate salinity-related problems (Daliakopoulos et al. 2016; Ismail et al. 2007).

Rice (*Oryza sativa* L.) is one of the most salt sensitive crops at seedling, early vegetative (Lutts et al. 1995) and at reproductive stages (Ismail et al. 2007; Singh et al. 2009). Salt causes two types of stress: osmotic stress with consequent cell dehydration and ion stress affecting cell ion homeostasis (Munns and Tester 2008). Both stresses disturbing metabolic processes, affecting tiller number, panicle length, spikelet number per panicle, negatively influence plant growth, development and yield (Kakar et al. 2019; Khatun et al. 1995; Munns and Tester 2008; Zeng and Shannon 2000). At every dS/m increase in soil electrical conductivity (EC) beyond the threshold value of 3.0 dS/m, the rice yield penalty is estimated approximately 12% (Grieve et al. 2012). Though salinity affects rice plants at all growing and developmental stages, its effect on young seedlings should be considered as highly detrimental since it directly influences plant establishment and eventually, yield. It is therefore relevant to develop varieties with salinity tolerance at the seedling stage that would promote an efficient growth at the early stages in salt affected soils, leading to healthy vegetative growth that can protect crop yield (Hoang et al. 2016).

Mechanisms of salinity tolerance in rice have been widely studied (Horie et al. 2012), but the genetic architecture of this trait is complex (Frouin et al. 2018). Several genes involved in rice salt tolerance have been studied (Reddy et al. 2017; Chuamnakthong et al. 2019) and numerous quantitative trait loci (QTLs) have been identified (Frouin et al. 2018; Negrao et al. 2011; Rahman et al. 2016; Mirdar Mansuri et al. 2020). Considering salt tolerance at early developmental stages, a major QTL named *Saltol* that accounts for 62–80% of phenotypic variation under salinity stress, has been identified in an inbred line population (RIL) obtained by crossing the salt tolerant *indica* landrace Pokkali and the salt sensitive *indica* cultivar IR29 (Bonilla et al. 2002; Thompson et al. 2010). The *Saltol* QTL is located on chromosome 1 and confers capacity to young rice plants growing under salt condition by maintaining in the shoots a low Na^+^/K^+^ molar ratio, which is essential for salt tolerance. The *OsHKT1;5* gene located in the *Saltol* region has been proposed to be the responsible gene for the salinity tolerance provided by *Saltol* (Kobayashi et al. 2017). *OsHKT1;5* encodes for a xylem-expressed Na^+^ selective transporter and acts by decreasing Na^+^ content in shoots and maintaining K^+^ homeostasis (Ren et al. 2005).

Salt-tolerant varieties at the seedling stage carrying the *Saltol* QTL include Nona Bokra, Pokkali and Hasawi (Platten et al. 2013), together with Capsule, a Bangladesh *indica* landrace (Rahman et al. 2016), FL478, a recombinant inbred line derived from the cross between the salt-tolerant variety Pokkali and the salt-sensitive IR29 (Walia et al. 2005), and IR64-*Saltol* (Ho et al. 2016). IR64-*Saltol* was developed at IRRI from a cross between IR64, a rice variety with high-yield that has been widely cultivated around the world, and FL478, used as donor of *Saltol* (Ho et al. 2016).

Functional validation of *Saltol* QTL has been achieved using traditional marker assisted breeding programs for varietal improvement in *indica* subspecies (Singh et al. 2018), as well as through transgenic technology by ectopically expressing *Saltol* QTL-related salinity-induced genes in rice. These last validations include the intermediate filament (*OsIF*) encoding gene differentially expressed in genotypes with or without the *Saltol* QTL (Soda et al. 2018) and the *Saltol* QTL-localized histone gene binding protein-1b (*OsHBP1b*) belonging to bZIP transcription factors (Das et al. 2019). Rice plants modified to express these two genes exhibited improved tolerance towards multiple abiotic stresses, thus reflecting the importance of the genes that make up the QTL.

Several reports of *Saltol* QTL introgression into high yielding *indica* rice varieties like IR64, BR11, BRRI Dhan 28, as well as Basmati rice varieties, indicate improvement of salinity tolerance at the seedling stage (Bimpong et al. 2016; Babu et al. 2017; Geetha et al. 2017; Gregorio et al. 2013; Guo and Ye 2014; Hasan et al. 2015; Huyen et al. 2012, 2013; Krishnamurthy et al. 2020; Nair and Shylaraj 2021; Yadav et al. 2020). Conversely, to our knowledge only in a very recent study (Bundó et al. 2022) the effects of the *Saltol* QTL introgression into *japonica* varieties have been evaluated. This study was designed to introgress the *Saltol* QTL from donor parent IR64-*Saltol* into the temperate *japonica* genetic background of two salt-susceptible rice varieties, Vialone Nano and Onice, using Marker Assisted Back-Cross (MABC) approach and thus further evaluate its effect on salt tolerance into temperate *japonica* rice genetic background.

## Materials and Methods

### Plant Materials

The used recurrent parents (RP) were Onice and Vialone Nano, two elite rice Italian varieties (*Oryza sativa* subsp. *japonica*). Vialone Nano is a temperate *japonica* medium grain variety grown in Italy since 1937; it was developed by crossing the Vialone variety with the reduced-height variety Nano. Onice is a temperate *japonica* long grain variety, registered in 2011. IR64-*Saltol* (*Oryza sativa* subsp. *indica*) was used as donor parent for *Saltol* QTL. IR64-*Saltol* was developed at IRRI from a cross between IR64, an elite *indica* line, and FL478, a breeding line carrying the *Saltol* QTL with very high level of seedling stage salt tolerance (Singh et al. 2018). FL478, derived from IR29 x Pokkali cross, contains < 1 Mb *Saltol* QTL DNA segment from the salt tolerant parent Pokkali, located on chromosome 1 between 10.6 and 11.5 Mb (Thompson et al. 2010). IR64-*Saltol* was derived from three rounds of marker assisted back-crosses targeting only the *Saltol* QTL on chromosome 1 (Ho et al. 2016). Seeds of the IR64-*Saltol* donor parent utilized in the present work were kindly provided by IRRI.

### Marker-Assisted Backcrossing Strategy

**Fig. 1:**
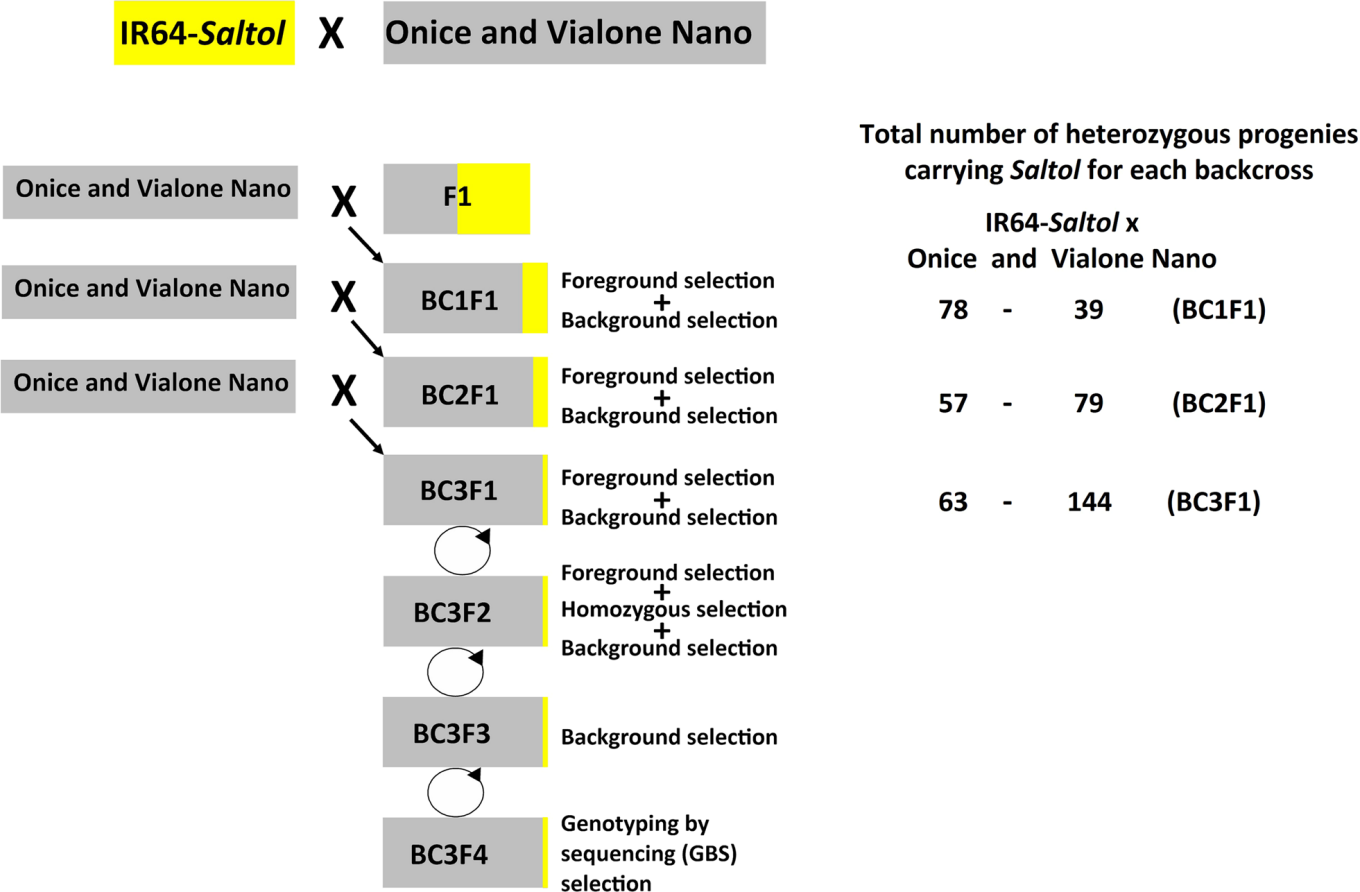
Flowchart of the Marker Assisted Back Cross (MABC) scheme used for *Saltol* QTL introgression in temperate *japonica* rice varieties Onice and Vialone Nano. The selected BC_3_F_4_ were used for field evaluations and hydroponic assays

To introgress the *Saltol* QTL into Onice and Vialone Nano, a marker assisted back-cross (MABC) program with foreground and background selections was applied. In the initial cross, Onice and Vialone Nano were employed as female, and IR64-*Saltol* as male. The hybridity of F_1_ plants was confirmed using the *Saltol* flanking SSRs markers, RM580 (IRGSP-1.0 position from bp 9,605,723 to 9,605,779) and RM493 (IRGSP-1.0 position from bp 12,281,143 to 12,281,320) (Additional file 2: Table S1). In the subsequent BC generations, until BC_3_F_1_, the respective RP were used as the female parents (Fig. 1). The SSR markers used to check the F_1_ plants were also used for the foreground selection of the BC_1_F_1_ plants. In the BC_2_F_1_ and BC_3_F_1_, the plant heterozygosity and the presence of the right allele at the *Saltol* locus were evaluated by using two KASP (Kompetitive allele specific PCR) markers id_1007745 (position 10,690,930 bp on chromosome 1) and K_id1008539 (position 12,591,394 bp on chromosome 1) flanking the *Saltol* locus. The same KASP markers were also used to select BC_3_F_2_ plants homozygous at the *Saltol* QTL.

For the background selection, about 240 KASP markers selected from the list of the 2000 validated rice markers established by the Integrated Breeding platform (https://www.integratedbreeding.net) were tested on the 3 parents and a set of 96 polymorphic markers were identified (Additional file 3: Table S2). As detailed below, these markers were used to assess the proportion of the recurrent parent genome recovery (RPGR%). Background selection was applied from the BC_1_F_1_ generation and the plants showing the highest RP% recovery (Table 2), were backcrossed to Onice and Vialone Nano to produce the subsequent BC generations, up to BC_3_F_1_. The selected BC_3_F_1_ plants were self-pollinated to produce BC_3_F_2_ seeds. Each BC_3_F_2_ plant was subjected to foreground selection to identify homozygous plants for both the foreground markers. The selected BC_3_F_2_ plants were subjected to background selection to assess those with the highest RP% recovery. The selected BC_3_F_2_ plants were finally self-pollinated to obtain BC_3_F_3_, which were screened in background selection to identify the “Best *Saltol* introgression lines”. Selected BC_3_F_3_ were self-pollinated to produce BC_3_F_4_ seeds that were finally genotyped with genotyping by sequencing (GBS) to precisely verify the level of homozygosity with respect to the recurrent parental genome (Fig. 1).

During the BCs, the MABC scheme was coupled to an embryo rescue (ER) technique to fasten the process. Briefly, 11 days after the cross the seeds under formation were harvested and deprived of glumes. Seeds were surface-sterilized by washing with 70% v:v ethanol for 1 min, followed by a washing with 30% v:v NaClO for 30 min and three rinses with sterile distilled water for 5 min. After this step, the embryos were harvested under sterile conditions and put on embryo rescue media (MS 0.5 ×) in jar for approximately 14 days at 26 °C for 14 h of light and 22.5 °C for 10 h in the dark. One liter media contained 2.2 g Murashige and Skoog medium, 20 g sucrose, 250 mg MES (4-Morpholineethanesulfonic acid, 2-(N-Morpholino) ethanesulfonic acid hydrate) and 2.5 g gelrite. Then, the germinated plantlets were acclimatized by transferring them in pots filled up with peat moss (1 L), vermiculite (0.55 L), calcium carbonate (1 g/L) and osmocote (3 g/L) and watered with tap water and kept at the same condition of temperature and light as above.

### Foreground and Background Selection

All genomic DNA extractions were performed by sampling young leaves of 15 days old plants and following the Mixed Alkyl Trimethyl Ammonium Bromide protocol (Risterucci et al. 2000). Ball bearings (3.0 mm) were used to crush frozen samples by shaking 30″ at a frequency of 30 rpm in a MM300 Mixer Mill (Retsch, Germany). The DNA was quantified on agarose gel and Qbit fluorimeter.

For SSR markers used for testing F_1_ and for foreground selection of BC_1_F_1_, the PCR was performed in a 10 μl reaction mix containing 20 ng genomic DNA, 0.04 µM forward primer, 0.2 µM reverse primer, 0.1 µM M13(FAM) primer 5′-CACGACGTTGTAAAACGAC-3′, 0.4 mM dNTPs mix, PCR buffer (5×), 1.5 mM MgCl_2_, and 0.5 U of Taq DNA polymerase. The PCR was run for 20 cycles with touch-down comprising of denaturation for 45″ at 94 °C, followed by annealing for 45″ at 61/51 °C decreasing by 0.5 °C every cycle, and primer elongation for 45″ at 72 °C. This was followed by 24 cycles comprising of denaturation for 45″ at 94 °C, annealing for 45″ at 51 °C, and primer elongation for 45″ at 72 °C, sandwiched between an initial denaturation for five minutes at 94 °C and the final extension for seven minutes at 72 °C. The amplified products were visualized by capillary electrophoresis using the Genome Analyzer 3130.

For foreground selection of BC_2_F_1_, BC_3_F_1_ and BC_3_F_2_ generations, KASP-based genotyping was performed in a 4 μl reaction consisting of 2 µl of genomic DNA [20 ng], 2 µl of KASP 2X Master Mix and 0.055 µl of KASP Assay supplied by LGC Genomics, and 0.032 µl MgCl_2_ 50 mM. The analyses were carried out using the 7500 Applied Biosystem instrument setting the allelic discrimination program in three steps: (1) pre-read step of 1′ at 60 °C; (2) amplification reaction consists of an hold of 15′ at 94 °C; 10 cycles of 20″ at 94 °C, 60″ from 61 to 55 °C in the touch-down method decreasing by 0.6 °C, 26 cycles of 20″ at 94 °C and 1′ at 55 °C; (3) post-read for 1′ at 40 °C. From BC_1_F_1_ generation onward, a set of 96 KASP markers, polymorphic between each recurrent parent *vs* IR64-*Saltol*, were run on chips on BioMark HD to assess the RP% recovery of each line. The chips were loaded following the standard protocol from Fluidigm. The PCR program had 3 steps: (1) 1800″ at 70 °C, 600″ at 25 °C and 900 s at 95 °C; (2) 1 cycle 10″ at 95 °C, 10″ at 57 °C and 10″ at 72 °C; 12 cycles 20″ at 94 °C and 60″ by touchdown from 65 to 57 °C with a cooldown final step at 20 °C. The fluorescence was read with Fluidigm SNP Genotyping Analysis software.

### Genotyping by Sequencing

Genomic DNA was extracted from young leaves of introgression lines and their respective parental lines by high-throughput automated methods using NucleoMag Plant kit (Macherey–Nagel) and its quality was verified on 1.5% agarose gel. After Hoechst quantification, DNA concentrations were normalized. DNAs were digested individually with the *ApeKI* restriction enzyme. GBS sequencing library was prepared by ligating the digested DNA to unique nucleotide adapters (barcodes) followed by PCR. Sequencing was performed using Illumina HiSeq3000. To detect informative SNPs, fastq files were analyzed using Tassel V5 pipeline and an alignment on the *O. sativa* Nipponbare reference genome (MSU7) with Bowtie2. Only polymorphic parental loci were kept and filtered on heterozygous rate (< 40%) and then imputed with Beagle v5.0. 15,580 imputed SNP were kept for analysis. The percentage in base pair (%bp, Table 3) was calculated by attributing to each SNP a base number value. Each value was calculated by dividing by 2 the difference of position between the SNP before and the SNP after. % bp is the sum of all donor’s SNP value by the total genome length.

### Agronomic Evaluations

BC_3_F_4_ seeds coming from seven Onice-derived lines and from six Vialone Nano-derived lines were evaluated together with the recurrent parents in field trials under non-salinized soil conditions to evaluate their main agronomic traits in reference agro-environments. The field experiments were set-up in two different sites, at the CREA rice research farm in Vercelli (45° 19′ 18″ N; 8° 22′ 06″ E) and at SIS rice farm in Malalbergo (Bologna) (44° 41′ 50″ N; 11° 28′ 53″ E) during 2019 season. Field plots of 9 m^2^ were organized in randomized blocks with three and two replicates in Vercelli and Malalbergo, respectively, and were managed according to common agricultural practices for rice cultivation in the area, with direct dry-seeding on May the 2nd and continuous flooding of paddy from the three-leaf stage until the end of ripening stage (Monaco et al. 2021). Flowering date, plant height, panicle length, seeds number per panicle, sterility and paddy yield were evaluated with subsamples collection in each plot following the methods described in Volante et al. (2017) for rice phenotyping.

### Hydroponic Assays of the Selected Lines and Parents

Rice seeds were surface-sterilized (3 min in 70% v:v EtOH, 30 min in 1.5% v:v NaClO and 0.02% Tween 20) and, after five rinses with sterile distilled water in sterile conditions, were placed on filter paper saturated with distilled water and incubated in the dark at 26 °C. Seven days later, seedlings were transferred into 10 L plastic tank (6 seedlings of each genotype in each tank) containing a modified Yoshida solution (1.43 mM NH_4_NO_3_, 0.51 mM K_2_SO_4_, 0.85 mM KH_2_PO_4_, 0.12 mM K_2_HPO_4_, 0.75 mM CaCl_2_ 2H_2_O, 1.64 mM MgSO_4_ 7H_2_O, 9.5 μM MnCl_2_ 4H_2_O, 0.075 μM (NH_4_)_6_ Mo_7_O_24_ 4H_2_O, 18.9 μM H_3_BO_3_, 0.15 μM ZnSO_4_ 7H_2_O, 0.16 μM CuSO_4_ 5H_2_O, 35.75 μM FeSO_4_-EDTA, pH 5) and kept for a 7-day pre-treatment period in a growth chamber maintained at 26 °C and 80% relative humidity during the 16 h light period and at 22 °C, and 70% relative humidity during the 8 h dark period. Photosynthetic photon flux density was 400 μmol m^−2^ s^−1^. At the end of the pre-treatment, plants were split into two groups and one was exposed for seven or 14-days to salt stress (80 mM NaCl in the nutrient solution), while the other was kept in control conditions. Aeration was applied to the solution both in the pre- and salt-treatments. All hydroponic solutions were renewed every three days to minimize nutrient depletion. Three tanks for each condition were used.

Electrolyte leakage (EL) analyses were carried out seven days after salt treatment as described by Campo et al. (2014). Briefly leaf blades from the two youngest enlarged leaves of each plant were cut in 1 cm segments and washed in Milli-Q water. Segments were incubated in 15 mL tubes at 25 °C for 2 h in 10 mL of Milli-Q water in a shaker, thus, without removing the segments, electro-conductivity of the solution (Ec1) was measured by a pH/mV meter (HI5221, HANNA instruments, Italy). After autoclaving the tubes containing the leaf segments for 20 min, Ec2 was measured and the EL was calculated as (Ec1/Ec2) × 100. The effect of salt treatment was evaluated calculating for each genotype the relative increase of EL in the treated samples with respect to the control ones. Three independent experiments were performed with three replicates from four plants each.

For Na^+^/K^+^ molar ratio evaluation, plants were harvested after seven days of salt treatment. To remove apoplastic Na^+^ and K^+^, roots were washed twice for 10 min at 4 °C in a 25 mM Rb_2_SO_4_ solution before dissecting the plant tissues (young leaves, old leaves, sheath and stem, and roots). Samples were oven-dried at 70 °C for 72 h, and the resulting weight (DW) was measured. Tissue samples were digested in Teflon tubes filled with 10 mL of 65% (v/v) HNO_3_ by a microwave digester system (MULTIWAVE-ECO, Anton Paar GmbH) by applying two-steps power ramps (Step 1: to 500 W in 10 min, maintained for 5 min—Step 2: to 1200 W in 10 min, maintained for 15 min). After 20 min cooling, the mineralized samples were transferred into polypropylene test tubes and diluted (1:40) with Milli-Q bi-distilled water (Millipore). Na^+^ and K^+^ were measured by Inductively Coupled Plasma-Mass Spectrometry (ICP-MS; Bruker AURORA M90 ICP-MS, Bruker Daltonik GmbH, Bremen, Germany). An aliquot of a 2 mg L^−1^ internal standard solution (^72^Ge, ^89^Y, ^159^Tb) was added to both samples and calibration standards to a final concentration of 20 µg L^−1^. Possible polyatomic interferences were removed by Collision–Reaction–Interface with an H_2_ flow of 70 mL min^−1^. Three independent experiments were performed (three replicates from three plants each). The stress injury score based on IRRI standard evaluation system (SES; IRRI 2013) was recorded on the same plants at both seven and 14 days after salt treatment. Shoot and root fresh weight (FW), dry weight (DW), as well as shoots and roots length were evaluated at the 14th day. Leaf relative water content (RWC) was evaluated according to Barrs and Weatherley (1962) at the end of the 14-d treatment period. Briefly, blades from the two youngest enlarged leaves from each plant were weighed (FW), dipped in distilled water in a Petri dish and kept in the dark for 24 h at 26 °C. Turgid weight (TW) was measured after gently patting dry the leaves with a paper towel. Dry weight was measured after 48 h oven drying at 60 °C. RWC was calculated as [(FW-DW)/(TW-DW)] × 100.

### Statistical Analysis

Field trials data were separately analyzed for each site and recurrent parent through ANOVA and Dunnett’s post-hoc test using R environment (R Core Team 2013).

Significant differences in RWC, S/R FW ratio, shoot and root length between plant grown in control or salt conditions were determined by Student’s t-test, evaluating pairwise comparisons of mean differences. Data concerning EL and Na^+^/K^+^ molar ratio were subjected to ANOVA analysis. The homogeneity of the variances was checked with a Levene’s test. A Tukey post-hoc test was carried out with Bonferroni correction, and significance was set at *p* < 0.05.

## Results

### Marker Assisted Introgression of *Saltol* into Onice and Vialone Nano

Assessment of hybridity of F_1_ plants and foreground selection of the BC_1_F_1_ plants was conducted using the *Saltol* flanking SSRs markers, RM580 and RM493 (Additional file 2: Table S1), while in the BC_2_F_1_ and BC_3_F_1_, the evaluation of plant heterozygosity and foreground selection were performed with the KASP markers id_1007745 and K_id1008539 flanking the *Saltol* locus.

**Fig. 2:**
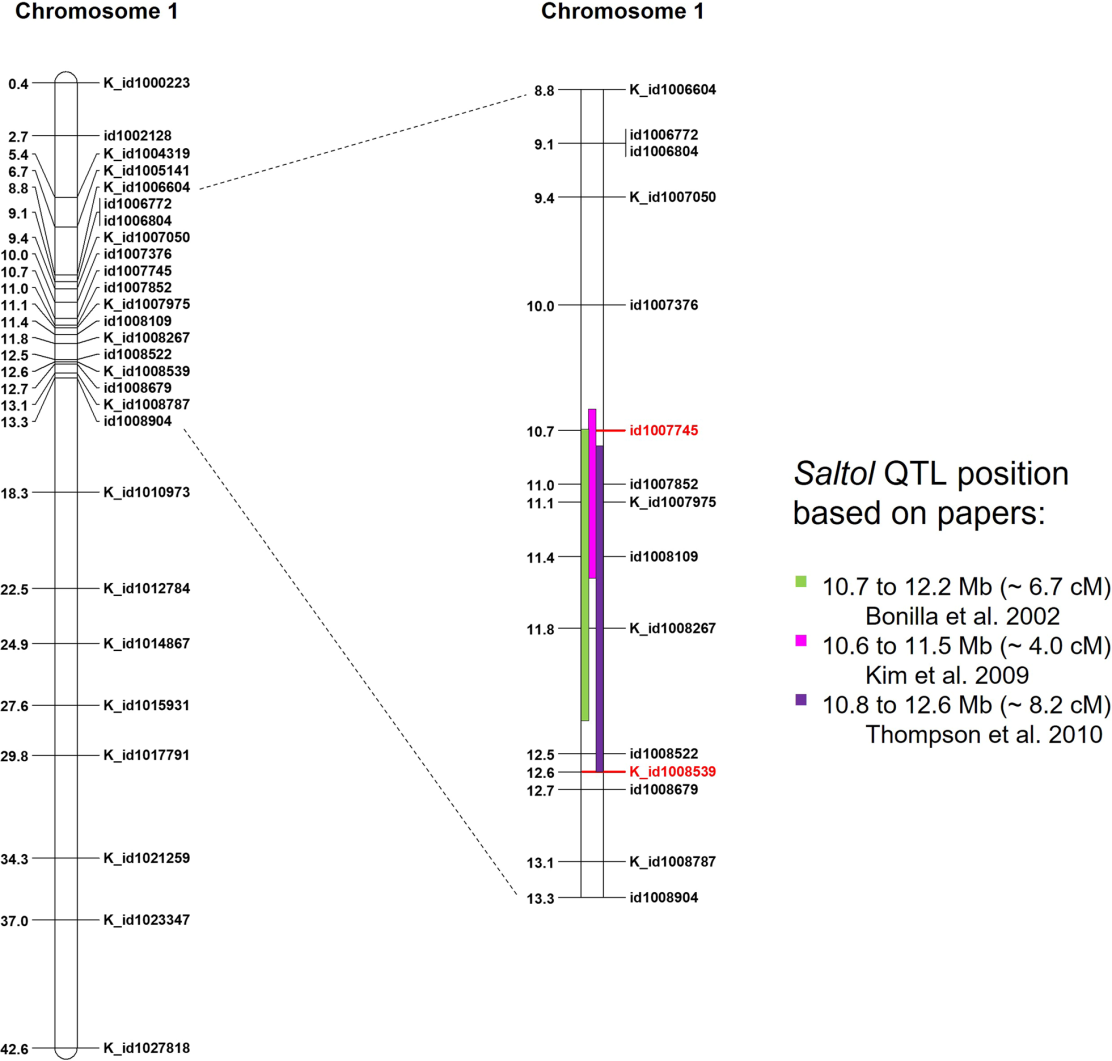
Physical position on the Nipponbare genome (Os-Nipponbare-Reference-IRGSP-1.0) of the 27 KASP markers for chromosome 1 utilized for the background selection (left image). Position of the markers is reported with respect to the position of the *Saltol* QTL in the indicated mapping studies; markers used for the foreground selection are highlighted in red (right image)

To perform the background selection, a panel of 96 KASP markers polymorphic between recurrent (Onice and Vialone Nano) and donor (IR64-*Saltol*) parents were selected. Among them, 92 markers were polymorphic between Onice and IR64-*Saltol*, while 86 were polymorphic between Vialone Nano and IR64-*Saltol*. The panel contained 27 markers localized on chromosome 1 and, among them, 7 were located within the *Saltol* region as defined by markers id1007745 and K_id1008539, bp 10,690,930 and bp 12,591,394 of the rice genome, respectively (Fig. 2; Additional file 3: Table S2). The remaining 69 markers were distributed on the other 11 chromosomes, with a range of 4–9 markers per chromosome (Table 1; Additional file 3: Table S2).

**Table 1:** Distribution of the KASP markers used in the background selection on the 12 rice chromosomes

Number of polymorphic KASP markers	Chromosome
27	Os01
7	Os02
8	Os03
4	Os04
5	Os05
9	Os06
7	Os07
8	Os08
5	Os09
4	Os10
7	Os11
5	Os12

To introgress the *Saltol* QTL, crosses between Onice and Vialone Nano, both temperate *japonica* rice varieties, and the *indica* variety IR64-*Saltol* were followed by backcrosses and selfing generations, as summarized in Table 2. A total of 550 BC_1_F_1_ plants were obtained for the two crosses; foreground selection yielded 117 plants heterozygous at markers RM580 and RM493 flanking the *Saltol* locus. After the background selection, 12 BC_1_F_1_ lines were selected for the Onice backcross scheme, with a RP% genome recovery between 46 and 72%, while 10 BC_1_F_1_ lines were selected in case of Vialone Nano (RPGR% between 45 and 61%). These lines were used as males for the subsequent round of backcrosses. BC_2_ generated 374 BC_2_F_1_ plants; foreground selection identified 131 plants heterozygous for markers id_1007745 and K_id1008539 flanking the *Saltol* locus. After the background selection, 17 BC_2_F_1_ plants were selected for Onice backcrosses, with RPGR% of 75–90%, and 18 BC_2_F_1_ plants for Vialone Nano backcrosses with RPGR% of 73–89%. These lines were used as males for the last backcross. BC_3_ yielded 518 BC_3_F_1_ plants; of these, 207 were identified as heterozygous for markers id_1007745 and K_id1008539. After the background selection, 29 plants were selected for selfing of which 14 from the Onice and 15 from the Vialone Nano backcrosses. Selected plants showed a RPGR% from 89 to 98%.

**Table 2:** Number of plants analyzed and RPGR% in back-cross generations during the marker assisted introgression of *Saltol* QTL

Generation	Tested and selected plants	Onice × IR64-*Saltol*	Vialone nano × IR64-*Saltol*
BC_1_F_1_	Tested plants	330	220
	Heterozygous plants at flanking markers	78	39
	Selected plants in background selection	12	10
	RPGR (Os01 excluded)	46–72%	45–61%
BC_2_F_1_	Tested plants	166	208
	Heterozygous plants at flanking markers	57	74
	Selected plants in background selection	17	18
	RPGR (Os01 excluded)	75–90%	73–89%
BC_3_F_1_	Tested plants	242	276
	Heterozygous plants at flanking markers	63	144
	Selected plants in background selection and selfed	14	15
	RPGR (Os01 excluded)	89–98%	91–98%
BC_3_F_2_	Tested plants	500	468
	Homozygous plants at flanking markers	119	99
	Selected plants in background selection and selfed	21	19
	RPGR (Os01 excluded)	92–100%	93–100%
BC_3_F_3_	Tested plants	133	144
	Homozygous plants at flanking markers	62	48
	Selected plants in background selection and selfed	14	12
	RPGR (Os01 excluded)	93–100%	94–100%
BC_3_F_4_	Tested plants	14	12
	Homozygous plants at flanking markers	14	12
	Selected and selfed plants	14	12

Tested plants indicate the number of analyzed plants, heterozygous/homozygous plants at flanking markers indicate the number of plants selected in foreground selection that have the marker alleles flanking *Saltol* locus in heterozygous or homozygous condition. RPGR% = 1 − (KASP heterozygous _(not included in Saltol locus)_ /total KASP markers)* 100

**Fig. 3:**
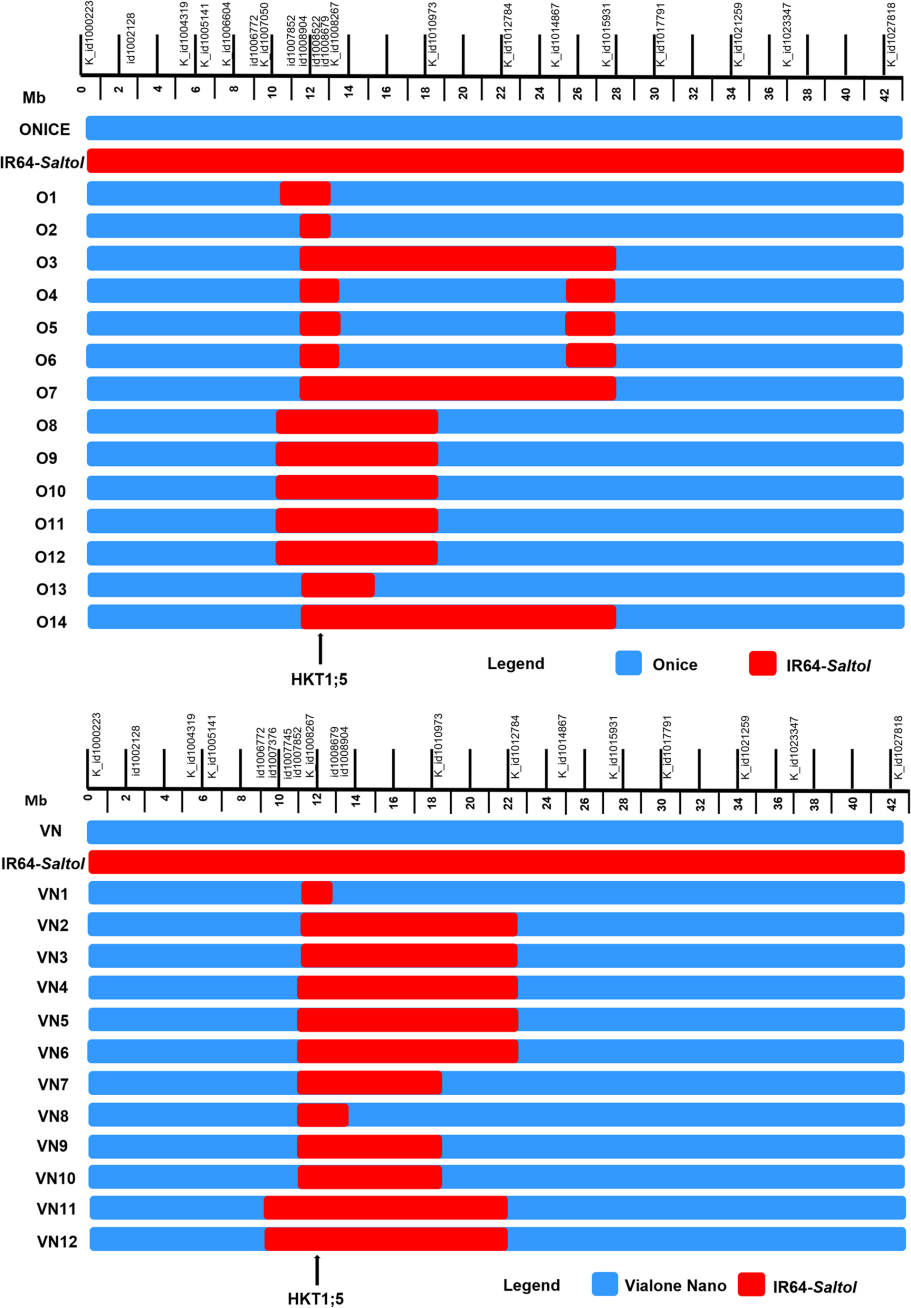
Graphical representation based on the 96 KASP marker alleles of Onice and Vialone Nano “Best introgression BC_3_F_4_ lines” (for Onice, from O1 to O14; for Vialone Nano, from VN1 to VN12) carrying *HKT 1;5 Saltol* QTL, depicting the extent of recovery of the carrier chromosome 1

Selfing of BC_3_F_1_ produced a total of 968 BC_3_F_2_ plants; foreground selection identified 218 plants (119 for Onice and 99 for Vialone Nano) homozygous for markers id_1007745 and K_id1008539. Then, the background selection carried out on these plants allowed the identification of 21 lines for Onice with RP% recovery between 92.4 and 100%, and 19 lines for Vialone Nano with RP% recovery between 93 and 100% (Table 2). An additional round of background selection was carried out on 277 BC_3_F_3_ plants (133 Onice lines and 144 Vialone Nano lines). This final step led to the selection of 14 lines for Onice and 12 lines for Vialone Nano as the best ones for percentage of recurrent parent genome recovery (Fig. 3, Additional file 4: Table S3). These selected lines were subjected to an additional selfing generation and foreground selection to obtain BC_3_F_4_ lines (Table 2).

**Table 3:** Ranking of BC_3_F_4_ lines for the RPGR% as indicated by the GBS results (% SNP donor) and comparison with performances of the 96 KASP markers panel

ID_GBS	% SNP donor*	% SNP recurrent	% bp from donor not detected by KASP markers
O2	1.06	98.94	1.04
VN1	1.03	98.97	1.03
VN8	1.85	98.15	1.11
O1	2.14	97.86	1.80
O5	2.57	97.43	1.50
O6	2.57	97.43	1.49
O10	2.94	97.06	0.44
O11	3.01	96.99	0.50
O8	3.21	96.79	0.70
O12	3.19	96.81	0.69
O9	3.17	96.83	0.67
VN4	3.14	96.86	0.00
O13	3.55	96.45	2.76
VN10	3.74	96.26	1.39
O4	3.91	96.09	2.83
VN12	3.78	96.22	0.56
VN7	4.61	95.39	2.26
VN9	4.56	95.44	2.21
VN11	4.16	95.84	0.48
VN2	4.16	95.84	1.35
VN3	4.16	95.84	1.36
VN6	4.73	95.27	1.60
VN5	4.73	95.27	1.59
O3	5.12	94.88	0.84
O7	6.34	93.66	2.06
O14	6.64	93.36	2.37

### Genotyping by Sequencing (GBS) of Selected BC_3_F_4_ Lines

To achieve a better characterization of the genomic landscape of the lines obtained by the MABC program, selected lines were further subjected to GBS using 15,580 SNP markers polymorphic between the *Saltol* donor IR64-*Saltol* and the recurrent parents Onice and Vialone Nano (Additional file 5: Table S4). For the lines deriving from Onice, the GBS analysis was applied to the 14 BC_3_F_4_ lines selected following the results of the forward and background selection. Similarly, for the lines derived from Vialone Nano, GBS was applied to the 12 BC_3_F_4_ lines.

**Fig. 4:**
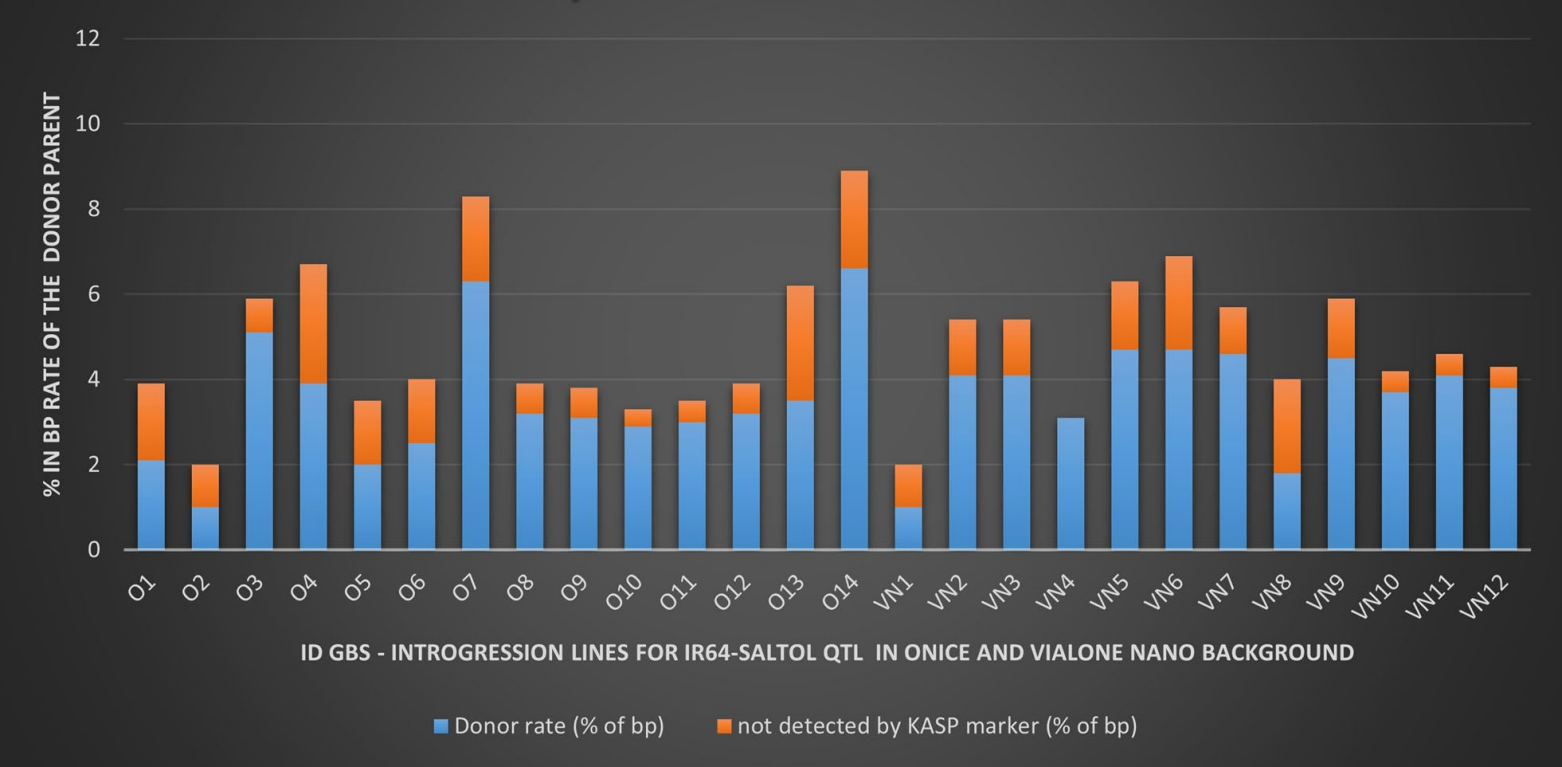
Comparison of the performances of 96 KASP marker panel and GBS in evaluating the RP genome recovery in the selected BC_3_F_4_ introgression lines. The blue bars represent the percentage value in bp of the donor rate based on the KASP markers used in the background selection, while the orange bars represent the percentage value in bp calculated on the GBS data but not detected by KASP markers

GBS analysis highlighted a RP% genome recovery ranging from 93.36 to 98.97% and the two best lines, O2 and VN1, showed a RPGR% of 98.94 and 98.97 for the Onice and Vialone Nano genomes, respectively (Fig. 4, Table 3). When recovery of the recurrent parent genomes obtained from GBS data were compared with those obtained from background selection made with the 96 KASP marker panel, an over-estimation of the RP% genome recovery was highlighted for the last procedure (Table 3).

### In-Field Evaluations of Lines

These field trials were carried out under non-salinized conditions with the purpose of identifying introgressed lines in which agronomical traits of the recurrent parents were maintained, to be then examined for salinity tolerance at the seedling stage. Towards this purpose, the best BC_3_F_4_ lines identified by background selection and GBS (7 Onice-derived lines and 6 Vialone Nano-derived lines) were tested in field trials in non-stress conditions and compared to their respective recurrent parents (Table 4). For Onice lines, yield data showed significant differences in Vercelli site for 1 out of 7 lines with a lower value (7.37 t ha^−1^) compared with the recurrent parent (10.57 t ha^−1^). Moreover, statistically significant differences among some lines and Onice recurrent parent were detected for plant height in both sites, and for panicle length, seed number and flowering time in Vercelli site. More precisely, 5 out of 7 new lines in Malalbergo and 2 out of 7 lines in Vercelli showed higher values of plant height compared with the recurrent parent, while 2 lines in Vercelli showed a delay in the flowering date. For Vialone Nano, significant differences were detected for plant height in Malalbergo and for flowering date in both sites, with 1 line out of 6 that anticipated flowering in Malalbergo and 5 lines which delayed it in Vercelli. After these agronomic evaluations in field conditions three lines (O1, VN1 and VN4), were selected as the most promising lines for commercial exploitation. These lines, which had shown high RP% genome recovery (Table 3), did not show significant differences from their respective recurrent parent for any of the phenotypic traits considered in the field trials (Table 4).

**Table 4:** The results and statistics of phenotypic evaluations of BC_3_F_4_ introgression lines and the recurrent parent Onice (A) and BC_3_F_4_ introgression lines and the recurrent parent Vialone Nano (B), tested in two experimental sites (Malalbergo and Vercelli). ANOVA and Dunnett’s test significant for p(F) < 0.05

Variable	Unit	Site	Total avg	ANOVA	Dunnett's test			O1/all lines*100 (index)^(b)^
				p(F)	Recurrent parent	O1 line^(a)^	LSD	
(A)								
Yield	Paddy 13% t ha^−1^	Malalbergo	6.21	0.075	6.46	6.76	1.18	109.6
		Vercelli	10.1	0.022*	10.6	10.6	2.94	104.2
Plant height	cm	Malalbergo	84.1	< 0.01**	74.3	74.3	7.97	86.9
		Vercelli	100.2	< 0.01**	94.5	92.7	10.7	91.8
Panicle lenght	cm	Malalbergo	13.8	0.146	13.5	13.2	3.57	95.0
		Vercelli	16.6	0.030*	16	16.1	3.36	97.0
Seed number	Number per panicle	Malalbergo	71.2	0.766	72.2	69.0	20.0	97.2
		Vercelli	92.8	< 0.01**	120.2	95.3	46.2	107
Flowering	Days from 1st July	Malalbergo	45.6	0.272	48.5	41.5	7.56	91.8
		Vercelli	46.2	< 0.01**	43.0	47.3	4.55	101.3
Sterility	Scale	Malalbergo	1.19	0.707	1.0	1.0	1.43	82.6
		Vercelli	1.13	0.410	1.0	1.0	1.09	87.7

Variable	Unit	Site	Total avg	ANOVA	Dunnett's test				VN1/all lines*100 (index)^(d)^	VN4/all lines*100 (index)^(e)^
				p(F)	Recurrent parent	VN1 line^(c)^	VN4 line^(c)^	LSD		
(B)										
Yield	Paddy 13% t ha^−1^	Malalbergo	6.26	0.09	6.42	6.08	6.35	1.82	97.1	101.4
		Vercelli	9.34	0.17	N.A.^(^^f)^	8.51	9.33	−	93.3	102.3
Plant height	cm	Malalbergo	121	0.046*	120	113	114	23.8	93.2	94.1
		Vercelli	132	0.21	132	136	129	13.6	102.9	97.6
Panicle lenght	cm	Malalbergo	19.7	0.29	21.2	19.5	19.8	3.21	100.5	102.1
		Vercelli	19.2	0.53	18.8	20.1	19.2	2.99	104.1	99.5
Seed number	Number per panicle	Malalbergo	120	0.87	128	110	117	51.2	92.4	98.3
		Vercelli	120	0.23	104	123	138	47.5	101.7	114.0
Flowering	Days from 1st July	Malalbergo	48.5	0.018*	48.5	49.0	50.5	6.38	101.0	104.1
		Vercelli	52.7	< 0.01**	50.0	55.7	54.0	2.97	104.3	101.1
Sterility	Scale	Malalbergo	1.1	0.5	1.0	1.0	1.0	0.89	92.6	92.6
		Vercelli	1.2	0.190	1.0	0.7	1.0	1.56	54.9	82.0

Asterisks indicate statistically significant differences (*p(F) < 0.05; **p(F) < 0.01)

^(a)^O1 introgressed line selected for the hydroponic assessment

^(b)^Average of O1 line/average of all Onice lines*100

^(c)^VN1 and VN4 introgressed lines selected for the hydroponic assessment

^(d)^Average of VN1 line/average of all Vialone Nano lines*100

^(e)^Average of VN4 line/average of all Vialone Nano lines*100

^(f)^*N.A.* not available

### Physiological Characterization of Introgression Lines

**Fig. 5:**
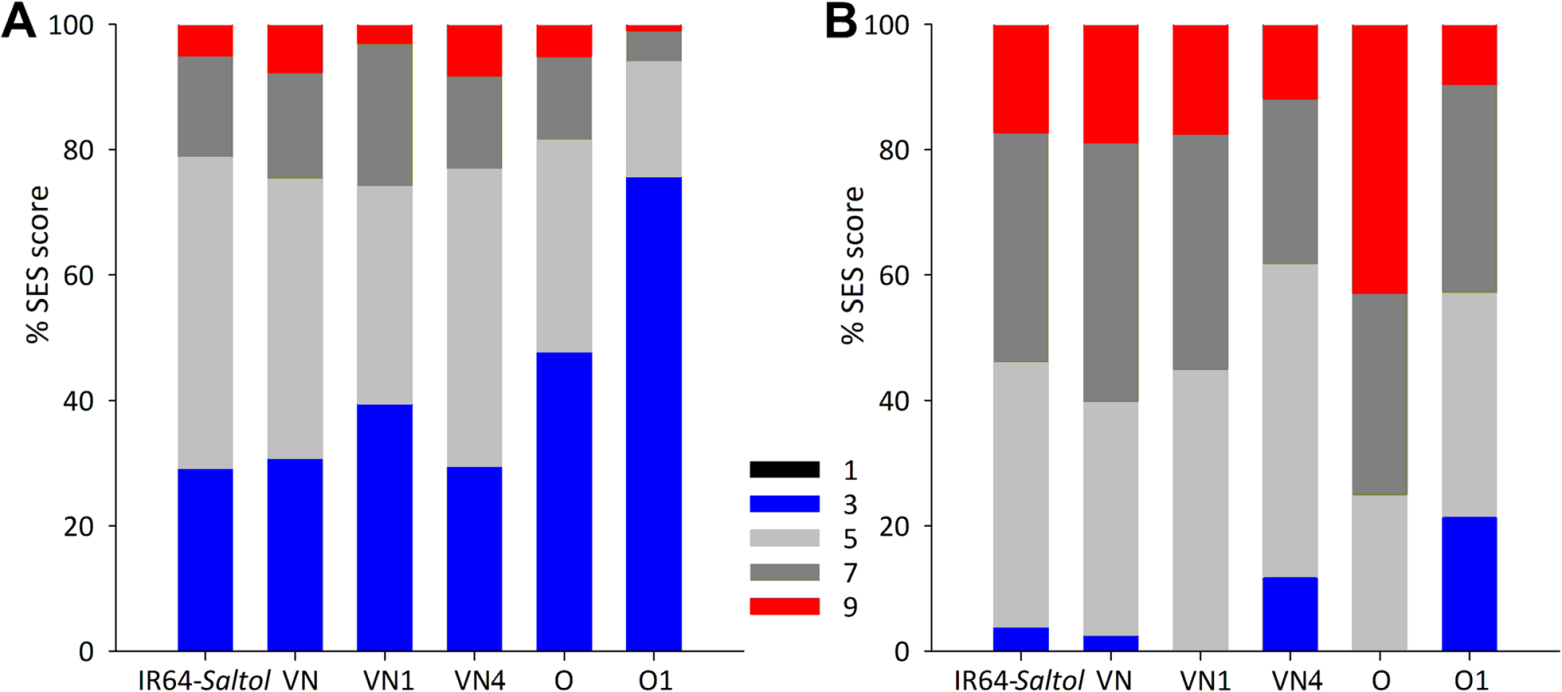
SES evaluation of the parental IR64-*Saltol*, the tolerant check, Vialone Nano (VN) and Onice (O), the susceptible checks and VN1, VN4, O1 introgression lines, after 7 days (**A**) and 14 days (**B**) of exposure to 80 mM NaCl. Data, expressed as the percentage of plants at each score value (1, highly tolerant; 3, tolerant; 5, moderately tolerant; 7, sensitive; 9, highly sensitive), are the means obtained from three independent experiments (12 plants/genotype each experiment)

A physiological characterization was carried-out on the three lines (O1, VN1 and VN4) selected after the agronomical field evaluations in comparison to Onice and Vialone Nano recurrent parents, the respective susceptible checks, and IR64-*Saltol,* the donor parent as well tolerant check. After seven days of salt stress (80 mM NaCl), most of the genotypes, including the tolerant donor parent IR64-*Saltol*, showed from 21 to 26% of plants with SES score values of 7–9 (Fig. 5A). However, in O1 line only less than 6% of the plants showed SES values ≥ 7. After 14 days of exposure to salt, the symptoms measured by the SES score worsened in all the genotypes (Fig. 5B). Nevertheless, for the VN4 and O1 lines about 62% and 57% of plants, respectively, were assigned to the tolerant (SES = 3) or moderately tolerant (SES = 5) group, while for their recurrent parents, Vialone Nano and Onice, only 40% and 25% of the plants, respectively, showed SES scores ranging from 3 to 5. Conversely, the SES score of VN1 did not differ significantly from Vialone Nano recurrent parent. Regarding the SES parameter, it is interesting to highlight that, under the experimental conditions here assayed, both VN4 and O1 resulted significantly more tolerant than *Saltol* donor line IR64-*Saltol*. The symptoms of salt stress injuries after 14 days of salinization on shoots of parental IR64-*Saltol*, Vialone Nano, Onice and VN1, VN4, O1 introgression lines are exemplified in Additional file 1: Fig. S1.

**Fig. 6:**
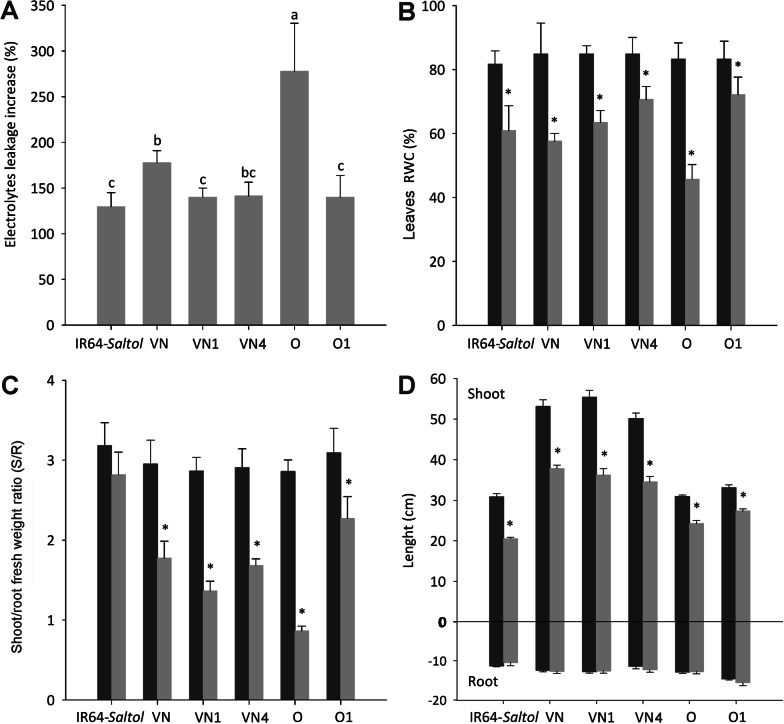
Salt stress response parameters evaluated in tolerant parent IR64-*Saltol*, susceptible parents Vialone Nano (VN), Onice (O) and VN1, VN4, O1 introgression lines. Plantlets were grown for 7 days (**A** Electrolyte leakage evaluation) or 14 days (**B** Relative water content in leaves; **C** shoot/root fresh weight ratio; **D** shoot and root length) in modified Yoshida solution in the absence (blue columns, controls) or presence (orange columns) of 80 mM NaCl. Data are the means from three independent experiments (12 plants per genotype for each experiment); bars represent the SE values (n = 36). Student’s t-test (*p < 0.05). Values with the same letter(s) are not significant at *p* < 0.05, as resulted by a Tukey post-hoc test with a Bonferroni correction

Salt conditions, already after seven days of exposure, provoked a marked increase in EL from the leaf cells of all the genotypes (Fig. 6A). In the sensitive recurrent parents Vialone Nano and Onice, the effects were particularly marked since EL raised by 178% and by 278%, respectively as compared with the controls. In the three introgression lines, the increase for EL values were similar to that of IR64-*Saltol* (+ 130%) and for VN1 and O1 resulted significantly lower than those measured in the sensitive parents.

Concerning RWC (Fig. 6B), while leaves of control plants showed similar values in the range of 82–85%, a significant decrease was recorded at 14 days in the plants exposed to salt. Under salinity, RWC reduction was lower in IR64-*Saltol* (− 19%) and in the introgression lines VN4 (− 17%) and O1 (− 13%) as compared with to the sensitive parents Vialone Nano (− 32%) and Onice (− 45%) that showed a RWC lower than 60%, indicative of a stress condition (Zhao et al. 2014).

Considering the shoot/root FW ratio (S/R; Fig. 6C), only O1 line under salt stress showed a reduction (− 26%) which was significantly lower than that of Onice recurrent parent (− 70%). The lowest reduction (− 11%) of S/R FW ratio was observed in IR64-*Saltol* donor parent. No significant effects of *Saltol* locus introgression were highlighted on shoot and root length (Fig. 6D).

In the blades of the young leaves of the three introgression lines and IR64-*Saltol*, Na^+^/K^+^ molar ratios were significantly lower than those of Vialone Nano and Onice recurrent parents (Table 5). A similar picture was observed also in the blades of old leaves even if in this tissue, the differences among the genotypes were less evident. Considering the whole shoot, significant differences were observed only for VN1, while for the other introgression lines, the Na^+^/K^+^ molar ratios were lower, but not significantly, than that of the recurrent parents, whereas high Na^+^/K^+^ molar ratios were observed in sheaths and stems. Concerning roots, only O1 line showed a Na^+^/K^+^ molar ratio significantly lower than that of the recurrent parent.

**Table 5:** Na^+^/K^+^ molar ratios in different tissues of VN1, VN4, O1 introgression lines and parental IR64-*Saltol*, the tolerant check, Vialone Nano (VN) and Onice (O), the susceptible checks

	Na^+^/K^+^ molar ratio				
	Blade		Sheath + stem	Shoot	Root
	Young leaves	Old leaves			
IR64-*Saltol*	1.28 ± 0.15^b^	2.07 ± 0.18^ab^	7.46 ± 0.80^a^	3.19 ± 0.62^a^	5.32 ± 0.19^a^
VN	1.81 ± 0.11^a^	2.29 ± 0.05^a^	4.06 ± 0.05^b^	2.92 ± 0.13^a^	1.46 ± 0.08^c^
VN1	0.44 ± 0.11^c^	1.29 ± 0.08^c^	1.89 ± 0.14^d^	1.68 ± 0.17^b^	1.68 ± 0.06^c^
VN4	0.87 ± 0.06^bc^	1.32 ± 0.02^c^	2.40 ± 0.19^d^	2.07 ± 0.21^ab^	1.07 ± 0.08^c^
ONICE	2.25 ± 0.12^a^	2.51 ± 0.13^a^	3.35 ± 0.20^c^	3.05 ± 0.24^a^	2.73 ± 0.11^b^
O1	1.00 ± 0.03^b^	1.71 ± 0.08^b^	3.60 ± 0.18^bc^	2.19 ± 0.22^ab^	1.29 ± 0.07^c^

Data are the means ± ES of three independent experiments (three replicates for three plants each). For each trait, values with same letter(s) are not significantly different at *p* < 0.05, as resulted by a Tukey post-hoc test with a Bonferroni correction

## Discussion

Rice is classified as the most salt sensitive cereal crop, particularly at seedling and reproductive stages (Kakar et al. 2019; Zeng and Shannon 2000). Its cultivation is particularly threatened by salt stress, which is currently worsened due to climate change that is causing rising of the sea level, thus bringing saline water to inlands and exposing rice growing areas to saline conditions. Since several rice cultivation areas in Italy and Europe, among which those located near river deltas, are currently or at risk of being exposed to salinity, a molecular breeding approach coupled with phenotypic evaluations under hydroponic conditions was adopted for the generation of salt tolerant introgression lines derived from crosses between the salt tolerant *indica* IR64-*Saltol* line and two salt sensitive *japonica* varieties.

The two *japonica* rice varieties used in this study, Vialone Nano and Onice, are of great economic importance in Italy for local and export markets, but both are susceptible to salt stress, e.g. Vialone Nano is reported as one of the most salt-sensitive rice Italian variety (Formentin et al. 2018). Introduction of salt tolerance into renowned rice varieties through conventional breeding method frequently implies that the developed progenies acquire unwanted traits due to linkage drag and possible negative effects on yield and grain quality traits of rice (Ismail et al. 2007; Thompson et al. 2010). Marker-assisted back-cross (MABC) is a simple and efficient methodology to introduce specific traits into otherwise popular varieties, and has been already used to select salt tolerant rice varieties through the introgression of *Saltol* locus both in the *indica* (Krishnamurthy et al. 2020; Yadav et al. 2020) and *japonica* (Bundó et al. 2022) genetic backgrounds.

In this work, the application of MABC coupled to foreground and background selection based on molecular markers yielded lines harboring *Saltol* locus and high RP% genome recovery in only three years with a minimal linkage drag. Several MABC-based works have used microsatellites in the foreground selection of the *Saltol* locus (Ganie et al. 2016; Krishnamurthy et al. 2020; Linh et al. 2012; Nejad et al. 2008; Yadav et al. 2020). We took advantage from a high-throughput genotyping method based on KASP markers, for both, foreground and background selections, performed during the MABC cycles. To maximize the recovery of recurrent background of the chromosome 1 carrying the *Saltol* QTL, 27 polymorphic KASP markers, including 7 markers within the 1.9 Mb QTL region, were used to assess the precise introgression of the *Saltol* locus from the donor IR64-*Saltol*. The MABC scheme adopted in the present work involved three backcrosses, that theoretically would allow a recovery of the RP genome of 93.75%. After five rounds of background selection, up to BC_3_F_3_ generation, lines with a final RPGR% ranging from 93 to 100%, as estimated of the basis of the KASP markers alleles were selected. Extension of the background selection also during the two selfing generations uncovered lines that became homozygous at additional loci for the RP alleles, thus increasing the success in the recovery of the recurrent parent genomes. Comparisons between the RPGR% estimated using the roughly 90 polymorphic KASP markers and 15,580 polymorphic SNPs obtained by GBS (Table 3), highlighted a slight under-estimation of the residual donor genome (estimated as % of bp of the donor) by the KASP marker evaluations, ranging from 0.44% (line O10) to 2.83% (line O4). GBS analysis was carried-out as a-posteriori verification to assess whether the number of polymorphic KASP markers that were used for the background selection (about 90) was appropriate to run an efficient marker-aided introgression. The 96 markers chip was considered a reasonable compromise between new need to scan the whole genome and the cost of each sample. Results obtained support that the regularly spaced 96 KASP markers employed in this work can effectively assess the recovery of the recurrent parent rice genomes during the background selection.

Effectiveness of the background selection was also confirmed by the results of the phenotypic evaluations in non-salinized field conditions, since most of the introgression lines did not differ significantly from their respective recurrent parents for the six phenotypic traits considered in at least one of the two experimental growing sites chosen for the trials, with the exceptions of the plant height for some Onice lines and flowering time for some Vialone Nano lines (Table 4). The field evaluation and the RP% genome recovery results ended with the selection of three lines, O1, VN1 and VN4, which did not show any significant difference with their respective recurrent parent for the investigated traits, showed high RP% genome recovery and were the most promising for commercial exploitation.

A physiological characterization was then conducted using salt tolerance assays under hydroponic conditions. Early effects of salt stress on tissues include the loss of selective permeability of the plasma-membrane, leading to a relative high leakage of electrolytes from the cell, as a symptom (Bajji et al. 2002; Demidchik et al. 2014). In addition, the ability of maintaining relatively low Na^+^/K^+^ molar ratios in the tissues photosynthetically more active under stress condition, confers tolerance to the plants and it is suggested to be the main physiological trait associated to *Saltol* QTL (Bonilla et al. 2002; Gregorio 1997; Gregorio et al. 2002; Waziri et al. 2016). In view of that, several physiological parameters, including low Electrolyte leakage (EL), the reduction in RWC, higher values of shoot and root fresh weight and shoot and root length (Ghosh et al. 2016), as well as low values of Na^+^/K^+^ molar ratios are used as indicators for relative salt tolerance in plants. The results obtained for the three selected lines (O1, VN1, and VN4), compared to the respective recurrent parents showed, as a whole, lower SES scores, reduced EL values, limited negative effects on both RWC and S/R fresh weight ratio, as well as a better ability to maintain relatively low Na^+^/K^+^ molar ratio in the blade leaves indicating an improved salinity tolerance. Nevertheless, the different lines exhibited different performances for the different parameters evaluated. The O1 line performed as the best for SES, leaf RWC (even better than IR64-*Saltol*) and shoot/root fresh weight ratio, while for the VN lines, VN4 performed slightly better than VN1 for SES (at 14 days of salt treatment), for leaf RWC and for the shoot/root fresh weight ratio. Variability in performances among introgression lines was also observed for the Na^+^/K^+^ molar ratios in the blades of young and old leaves, where all the lines performed better than IR64-*Saltol*, while for the whole shoot, a positive effect was observed only for VN1.

This variability in the response to salinity could be attributed to the complexity of salt tolerance character. Indeed, the *Saltol* QTL comprises several genes potentially contributing to salt tolerance including membrane transporters, signal transducers, transcriptional factors (Bundó et al. 2022; Chen et al. 2021; Das et al. 2019; Nutan et al. 2017, 2020). Additionally, it is also important to underline that despite the *Saltol* QTL has been extensively employed for improvement of salinity tolerance in rice breeding, the specific loci in *Saltol* responsible for salinity tolerance is still a matter of debate (Li et al. 2018; Walia et al. 2005). Currently, a role in conferring salinity tolerance at the seedling stage has been demonstrated only for *OsHKT1;5* located in the *Saltol* QTL (Kobayashi et al. 2017) and to the transcription factor *OsGATA8*, also localized within the *Saltol* QTL (Nutan et al. 2020).

Intra-QTL recombination could cause differential response of introgression lines carrying *Saltol.* In this work, the 1.9 Mb QTL region was monitored with seven polymorphic markers, on average one marker every 271 Kb, and recombination events were observed within the 1.9 Mb QTL region (Fig. 3; Additional file 4: Table S3), supporting that intra-QTL recombination can occur between *Saltol* flanking markers and the QTL region. The introgression line VN1 showed the Vialone Nano allele at the distal flanking marker id1007745, while still maintaining the IR64-*Saltol* haplotype at markers flanking the *OsHKT1;5* locus (Additional file 4: Table S3), as confirmed by the GBS data (Additional file 5: Table S4). However, the role of *Saltol* in salt tolerance was mainly ascribed to the presence of *OsHKT1;5*, candidate gene responsible to confer the capacity in maintaining good Na^+^/K^+^ homoeostasis (Kobayashi et al. 2017; Ren et al. 2005;). Concerning this aspect, the effectiveness of the introgression procedure adopted was confirmed, as all the three selected lines proved more efficient than the recurrent parents, as well as than *Saltol* donor, in maintaining a good ionic balance in the active photosynthetic tissues (Table 5).

The effectiveness of the *Saltol* region might also be affected by the genetic background of the recipient parents. The background effect could be ascribable to unknown interactions of *Saltol* locus with the genomic regions of RP or to the existence of other minor QTLs and their interaction with *Saltol* to provide effective seedling stage salinity tolerance in the donor parent (Babu et al. 2017; Singh et al. 2018). In several studies, indeed, no significant differences in salt tolerance responses could be observed in *Saltol*-containing rice lines with respect to lines without *Saltol* (Alam et al. 2011; De Leon et al. 2017; Han et al. 2020; Ho et al. 2016; Yadav et al. 2020).

## Conclusions

In this work the *Saltol* locus was successfully transferred to two elite Italian varieties by marker assisted backcross in a time frame of three years. The application of background selection until BC_3_F_3_ allowed the selection of lines with a RP genome recovery up to 98.97% based on 15,580 polymorphic SNP markers. Physiological evaluations for the three selected introgression lines obtained from the MABC procedure indicate an improved salinity tolerance at the seedling stage. The best introgression lines are currently under the path for their release for commercial production; in addition to this exploiting avenue, they can be used as elite *japonica* germplasm donors in breeding programs to transfer the salt tolerance phenotype to other *japonica* varieties. Finally, the introgression lines can be also used in pyramiding programs, where it will be possible to accumulate traits for salt tolerance at seedling stage together with traits affecting salt tolerance at other phenological stages, thus combining different salt tolerance QTLs in a single genetic background (Frouin et al. 2018).

## Supplementary Information

**Additional file 1. Fig. S1.** Visual symptoms of salt stress injuries (80 mM NaCl) after 14 days of salinization on shoots of parental IR64-*Saltol*, Vialone Nano, and Onice and the introgression VN1, VN4, O1 lines. Results from a representative experiment are shown.

**Additional file 2. Table S1.** *Saltol*-linked SSR markers, used for foreground selection on F_1_ and BC_1_F_1_ generations and their physical position on chromosome 1; primer nucleotide sequences, and physical locations within the *Saltol *QTL are provided.

**Additional file 3. Table S2.** List of the 96 polymorphic KASP markers used for the background selection. Chromosomal position (in bp), alleles of the KASP markers in the recurrent parents (Onice and Vialone Nano) and in the *Saltol* donor line (IR64-*Saltol*) are shown.

**Additional file 4. Table S3.** Diagrammatic representation of the KASP marker alleles in the selected Onice-derived (from O1 to O14) and Vialone Nano-derived (from VN1 to VN12) BC_3_F_4_ introgression lines.

**Additional file 5. Table S4.** Diagrammatic representation of the SNP marker alleles derived from GBS in the selected Onice-derived (from O1 to O14) and Vialone Nano derived (from VN1 to VN12) BC_3_F_4_ introgression lines.

## Data Availability

All data generated or analysed during this study are included in this published article [and its supplementary information files].

## Abbreviations

MABC: Marker-assisted backcross
GBS: Genotyping by sequencing
VN: Vialone Nano
O: Onice
KASP: Kompetitive allele specific PCR
SES: Standard evaluation system
EL: Electrolyte leakage
RWC: Relative water content
EC: Electrical conductivity
SSR: Single sequence repeat
FW: Fresh weight
DW: Dry weight
TW: Turgid weight
RP: Recurrent parent
ER: Embryo rescue
RPGR: Recurrent parent genome recovery
