# Supplementary material for: Marker-Assisted Introgression of the Salinity Tolerance Locus Saltol in Temperate Japonica Rice
Source: Rice (N Y). 2023 Jan 12;16:2. doi: 10.1186/s12284-023-00619-2 (PMC9837369; doi:10.1186/s12284-023-00619-2)
Supplement: Supplementary file 1 — Additional file 1. Fig. S1. Visual symptoms of salt stress injuries (80 mM NaCl) after 14 days of salinization on shoots of parental IR64-Saltol, Vialone Nano, and Onice and the introgression VN1, VN4, O1 lines. Results from a representative experiment are shown. [file 12284_2023_619_MOESM1_ESM.pdf]

Additional file 1: Supplemental Figure

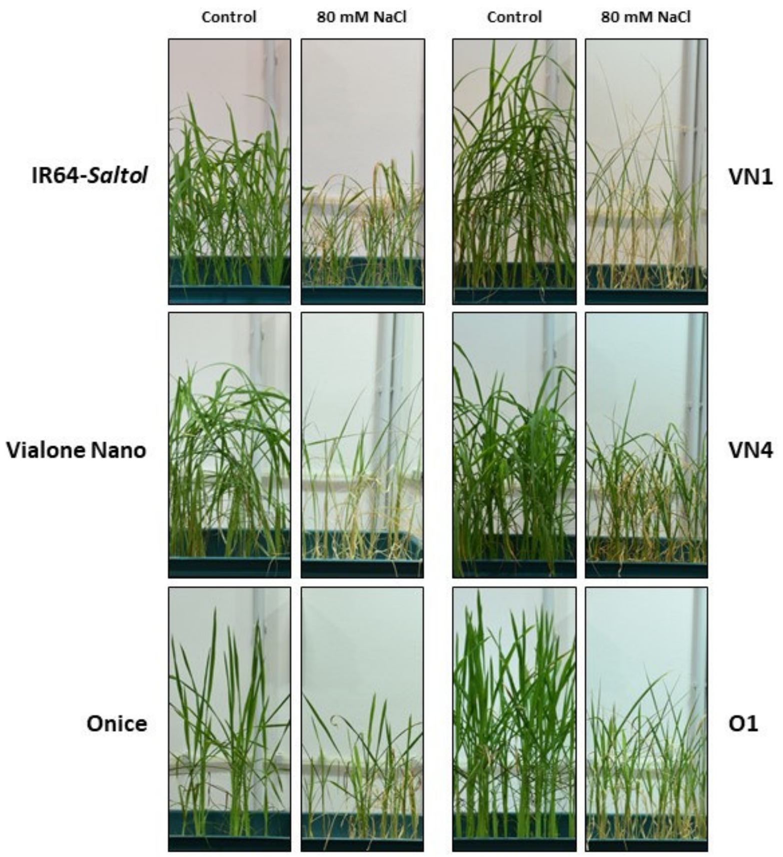

Fig. S1 Visual symptoms of salt stress injuries (80 mM NaCl) after 14 days of salinization on shoots of the tolerant donor parent IR64-*Saltol*, susceptible recurrent parents Vialone Nano and Onice, and VN1, VN4, O1 introgression lines. Results from a representative experiment are shown.
